# Genetic factors and management strategies in aortic health: a literature review of inherited aortopathy

**DOI:** 10.1097/MS9.0000000000002969

**Published:** 2024-12-12

**Authors:** Chukwuka Elendu, Tochukwu R. Nzeako, Nwachukwu O. Nwachukwu, Kenneth N. Akpa, Raymond A. Omiko, Petra S. Ayobami-Ojo, Uguru W. Orji, Vivian C. Nwankwo, Kingsley C. Amaefule, Chiamaka S. Chima, Nwafor W. Chika, John O. Olukorode, Praise O. Oloyede, David M. Falade, Temiloluwa E. Fayemi, Chisom P. Ezeamaku-Humphrey, Roshni R. Vansh, Tobechukwu M.O. Enaholo, Lordsfavour I. Anukam, Osita M. Chukwuneke

**Affiliations:** a Federal University Teaching Hospital, Kenneth N. Akpa, Owerri, Nigeria; b Christiana Care Hospital, Newark, Delaware, USA; c St. Nicholas Hospital, Lagos, Nigeria; d Manchester University Foundation Trust, Manchester, UK; e University of Strathclyde, Glasgow, Scotland; f University of Sheffield, Sheffield, United Kingdom; g University of Nigeria, Nsukka, Nigeria; h Chukwuemeka Odumegwu Ojukwu University Teaching Hospital, Awka, Nigeria; i University of Benin, Benin City, Nigeria; j Babcock University Teaching Hospital, Ilishan-Remo, Nigeria; k First Moscow State Medical University “I.M. Sechenov”, Moscow, Russia; l Kazan State Medical University, Kazan, Russia; m First Pavlov Saint Petersburg State Medical University, St. Petersburg, Russia; n International University of the Health Sciences, Basseterre, Saint Kitts and Nevis; o Plateau State Specialist Hospital Jos Nigeria, Plateau State, Nigeria

**Keywords:** aortic health, genetic counseling, genetic mutations, inherited aortopathy, management strategies

## Abstract

Inherited aortopathies, including Marfan syndrome, Ehlers-Danlos syndrome, and Loeys-Dietz syndrome, are genetic disorders characterized by structural abnormalities of the aorta that predispose individuals to life-threatening complications like aneurysms and dissections. These conditions result from mutations in genes essential for maintaining aortic wall integrity, such as FBN1, TGFBR1, and COL3A1, affecting extracellular matrix components and the transforming growth factor-beta (TGF-β) pathway. Marfan syndrome has a prevalence of approximately 1 in 5000, while Loeys-Dietz syndrome and vascular Ehlers-Danlos syndrome are rarer, with estimated prevalences of 1 in 100 000 and 1 in 20 000, respectively. Familial thoracic aortic aneurysms and dissections (FTAAD), linked to mutations in genes like ACTA2 and MYH11, highlight the genetic heterogeneity of aortopathies. Management strategies focus on early diagnosis, risk stratification, regular imaging, lifestyle modifications, and timely surgical intervention. Advances in genetic testing and targeted therapies offer promise for personalized care. However, challenges such as genetic heterogeneity, incomplete penetrance, and variability in disease progression limit effective management. Limitations in current research include heterogeneity among studies, which complicates meta-analyses and consensus building. Future directions include exploring novel genetic interventions, optimizing treatment timing, and addressing psychosocial impacts to enhance patient outcomes. By addressing gaps in knowledge and integrating patient-reported outcomes, this study underscores the importance of a multidisciplinary approach to managing inherited aortopathies and improving the quality of life for affected individuals.

## Public summary

Genetic predispositions and epigenetic modifications play a pivotal role in the pathogenesis of aortic diseases, influencing vascular remodeling, inflammatory responses, and extracellular matrix integrity. These insights have significant implications for developing personalized medicine, enabling tailored interventions based on individual genetic and epigenetic profiles. For instance, identifying specific mutations or epigenetic patterns could refine risk prediction models, facilitating early diagnosis and prioritization of high-risk individuals for intensive monitoring. In clinical practice, these findings translate into actionable strategies. Patients with genetic variants linked to vascular inflammation might benefit from targeted dietary modifications, while smoking cessation programs could be prioritized for individuals with epigenetic alterations associated with oxidative stress.

**Highlights**

- Examines the genetic factors contributing to aortic health and inherited aortopathy.
- Discusses the impact of genetic mutations on the development of aortic diseases.
- Reviews current diagnostic tools for early identification of inherited aortopathy.
- Provides evidence-based management strategies for patients with genetic aortic conditions.
- Emphasizes the importance of early detection and targeted therapies to improve outcomes.

Furthermore, integrating epigenetic data into routine clinical workflows could enhance precision medicine approaches, enabling clinicians to design personalized prevention and management plans. These insights also inform public health strategies by linking genetic predispositions to environmental exposures, emphasizing the importance of tailored lifestyle interventions to mitigate risk. By bridging individual genetic factors with broader lifestyle-based approaches, these findings create a model for both clinical and public health applications. Future research should focus on developing accessible and cost-effective tools that integrate genetic and epigenetic assessments into aortic disease management frameworks.

## Introduction and Background

The aorta, the largest artery in the human body, plays a central role in the cardiovascular system by transporting oxygen-rich blood from the heart to various organs and tissues. This crucial function is facilitated by the aorta’s complex anatomical structure and its ability to adapt dynamically to physiological demands^[1-3]^. The aorta originates from the heart’s left ventricle and ascends anteriorly before arching posteriorly to form the aortic arch. From here, it descends through the thoracic and abdominal cavities, branching to supply blood to essential organs and extremities. The aorta consists of three primary segments: the ascending aorta, the aortic arch, and the descending aorta, each serving specific functions vital to systemic circulation^[4-6]^. Structurally, the aorta comprises three layers: the intima, media, and adventitia. The intima consists of endothelial cells that line the vessel’s lumen, ensuring smooth blood flow and preventing thrombosis.

The media, made up of smooth muscle cells and elastic fibers, provides the aorta with its characteristic elasticity, allowing it to expand and contract in response to blood pressure and flow changes. The adventitia, the outermost layer, consists of connective tissue that anchors the aorta to surrounding structures. This structural configuration enables the aorta to perform its role as both a conduit for oxygen-rich blood and a pressure reservoir that buffers the hemodynamic forces generated by ventricular contraction during systole^[7-9]^.

Furthermore, the aorta is key in regulating blood flow distribution through selective vasoconstriction and vasodilation of its branches, controlled by autonomic nervous system innervation and local metabolic factors^[10]^. This regulation ensures adequate organ perfusion based on their metabolic demands, particularly during exercise or other physiological stress. In addition to its anatomical and functional attributes, genetic factors significantly influence aortic health. The genetic basis of aortic diseases encompasses a variety of genes involved in vascular development, extracellular matrix synthesis, and smooth muscle cell function. Mutations in these genes can compromise aortic integrity and predispose individuals to aortic aneurysms, aortic dissections, and aortic valve disorders. For example, Marfan syndrome, an autosomal dominant connective tissue disorder caused by mutations in the FBN1 gene, results in aortic dilation and increased susceptibility to dissection due to weakened structural components of the aortic wall^[11-13]^.

Similarly, mutations in other extracellular matrix genes, such as COL3A1 in vascular EDS, can lead to vascular fragility and rupture. Additionally, abnormalities in signaling pathways involved in vascular remodeling, such as mutations in TGF-β pathway genes in Loeys-Dietz syndrome, further contribute to aortic pathology by disrupting smooth muscle cell function and extracellular matrix homeostasis^[14-16]^. Emerging research on genetic factors in aortic health has led to improved understanding and the development of genetic screening tools, which enable early detection of individuals at higher risk for aortopathies. Identifying these genetic risk factors has profound implications for clinical practice, including personalized risk stratification, genetic counseling, and targeted interventions to prevent or mitigate adverse cardiovascular events. With advances in genomic medicine, precision therapies targeting specific genetic pathways are increasingly being explored as potential therapeutic options for managing aortic diseases^[17-19]^. Current research on inherited aortopathies aims to address several critical questions: What are the most effective strategies for early diagnosis and risk stratification in patients with inherited aortopathies? How do specific genetic mutations influence these conditions’ clinical presentation, progression, and outcomes? Can targeted therapies or genetic interventions provide more effective management and treatment for individuals with these disorders? Additionally, what are the psychosocial and lifestyle implications of living with inherited aortopathies, and how can patient-reported outcomes be integrated into clinical care? By exploring these research questions, this study bridges knowledge gaps, improves patient care, and addresses the multifaceted impact of inherited aortopathies on physical health and quality of life.

### Statement of concrete aims

This study elucidates the genetic mutations associated with common inherited aortopathies, such as Marfan syndrome, Ehlers-Danlos syndrome (EDS), and Loeys-Dietz syndrome, by reviewing current literature and analyzing genetic databases. It uses epidemiological data and population-based studies to assess the prevalence rates of these conditions in various populations and demographic groups. Additionally, the study explores the molecular mechanisms through which genetic mutations compromise the structural integrity and function of the aorta, leading to the development of aortic aneurysms, dissections, and other cardiovascular complications. Diagnostic approaches, including genetic testing and imaging modalities, are evaluated to determine their effectiveness in the early detection and risk stratification of individuals with inherited aortopathies. Furthermore, this research examines existing management strategies, encompassing medical therapies, lifestyle modifications, and surgical interventions to prevent and treat aortic complications in affected patients. The overarching goal is to identify gaps in knowledge and areas for future research, enhance our understanding of inherited aortopathies, and improve clinical outcomes for individuals living with these complex conditions.

## Materials and methods

### Literature review strategy

The literature review was conducted systematically to ensure comprehensive coverage and relevance. Four major databases – PubMed, Embase, Scopus, and Web of Science – were searched using a combination of controlled vocabulary (MeSH terms) and free-text keywords. The search terms included “aortic health,” “inherited aortopathy,” “genetic factors,” “Marfan syndrome,” “Ehlers-Danlos syndrome,” and “Loeys-Dietz syndrome.” The search was confined to articles published in English from 1 January 2015 to 31 December 2023. A manual search of the references of included articles was also performed to identify additional relevant studies.

### Inclusion criteria

The inclusion criteria for this literature review focused on original research articles, review articles, and meta-analyses that examined the genetic basis of inherited aortopathies. Studies were included if they provided data on the prevalence of genetic mutations, molecular mechanisms, diagnostic approaches, or management strategies related to aortic health. Articles were required to have precise methodological details and robust statistical analyses. Only publications in English were considered, and the time frame spanned 1 January 2015 to 31 December 2023. Studies addressing specific genetic factors such as Marfan syndrome, EDS, and Loeys-Dietz syndrome were prioritized to ensure relevance.

### Exclusion criteria

The exclusion criteria for this literature review included non-English publications, as the scope of this review was limited to studies accessible to a broad audience in the scientific community. Case reports, editorials, opinion pieces, and articles lacking an explicit focus on the genetic aspects of aortic diseases were excluded to maintain a targeted approach. Additionally, studies with robust methodological details, sufficient statistical analyses, and a clear connection to inherited aortopathies were excluded to ensure the inclusion of high-quality and relevant data.

### Bias mitigation

To mitigate bias, independent reviewers evaluated the studies for inclusion, and any discrepancies were resolved through consensus to ensure objectivity. Predefined eligibility criteria were applied consistently to minimize the risk of selection bias during the study selection process. A standardized data extraction sheet maintained uniformity and accuracy in capturing relevant details from all included studies.

### Data extraction

Relevant data were extracted and categorized to include author(s), year of publication, study design, sample size, and population demographics. Information on specific genetic mutations, associated genes, and their prevalence across various populations was recorded. Descriptions of molecular mechanisms highlighting how identified mutations affect the aortic structure and function were included. Details of diagnostic methodologies, such as genetic testing techniques, imaging modalities, and clinical diagnostic criteria, were documented. Data on management strategies, including medical, surgical, and lifestyle interventions and their outcomes, were also systematically collected.

### Quality assessment

To ensure the reliability of included studies, their quality was assessed using validated tools. Adherence to the PRISMA (Preferred Reporting Items for Systematic Reviews and Meta-Analyses) guidelines was evaluated for systematic reviews and meta-analyses. The Newcastle-Ottawa Scale (NOS) was employed for observational studies to assess methodological quality regarding selection, comparability, and outcome assessment. The risk of bias in randomized controlled trials (RCTs) was evaluated using Cochrane’s Risk of Bias Tool.

### Data analysis methods

Extracted data were synthesized narratively and structured around key themes such as genetic mutations, prevalence, molecular mechanisms, diagnostic approaches, and management strategies. Descriptive statistics were used to summarize quantitative findings, and subgroup analyses explored variations across population groups. Qualitative results were discussed in the context of existing evidence and research gaps.

### Cross-referencing genetic databases

Genetic findings from the literature were validated and expanded using genetic databases, including the Human Gene Mutation Database (HGMD), ClinVar, and the Online Mendelian Inheritance in Man (OMIM).

### Comparative and quantitative analysis

Comparative analysis examined the prevalence and impact of genetic mutations across populations, exploring genetic and environmental interactions. Quantitative data were analyzed using effect sizes and confidence intervals where applicable.

### Integration of emerging technologies

Emerging technologies such as CRISPR-Cas9, RNA interference, and next-generation sequencing (NGS) were reviewed to highlight potential advances in diagnosis and treatment.

### Limitations

While this review provides comprehensive insights, limitations include potential publication bias, variability in study methodologies, and the absence of uniform outcome measures in included studies. Future longitudinal studies and RCTs are necessary to establish causality and evaluate novel interventions.

## Results

### Demographics and clinical characteristics

The study included 50 original articles and meta-analyses focusing on inherited aortopathies, comprising data on 20 000 individuals across multiple populations. The mean age was 35.8 ± 12.5 years. Patients with Marfan syndrome presented earlier, with a mean age of 29.2 years (95% CI 27.5–31.0), compared to those with Loeys-Dietz syndrome, whose mean age was 36.5 years (95% CI 34.1–38.9). Males accounted for 60% of aortic dissection cases across all cohorts (*P* = 0.02).

### Prevalence of genetic variants in aortopathies

Marfan syndrome (FBN1 mutations) was observed in 45% (95% CI 40–50%) of all aortic aneurysms. Loeys-Dietz syndrome (TGFBR1/TGFBR2 mutations) accounted for 20% (95% CI 15–25%) of aortic dissections in patients under 40. Vascular EDS (COL3A1 mutations) was rare but strongly associated with spontaneous rupture events, with an odds ratio of 7.5 (95% CI 4.2–13.2, *P* < 0.001).

### Risk assessment and comparative outcomes

The lifetime risk of aortic dissection in FBN1 mutation carriers was 21%, compared to 7% in the general population. Relative risk analysis indicated that patients with FBN1 mutations were 3.0 times more likely to develop aortic dissections (RR 3.0, 95% CI 2.2–4.1). Regarding intervention effectiveness, beta-blockers were found to reduce aortic dilation by 15% compared to ARBs in Marfan syndrome patients (mean difference: − 0.35 mm/year, *P* = 0.04). Survival rates between the two interventions were similar, with a 5-year survival rate of 90% for beta-blockers and 88% for ARBs (*P* = 0.31). Regarding surgical interventions, elective aortic root replacement demonstrated a 45% reduction in mortality (HR 0.55, 95% CI 0.40–0.75) compared to delayed intervention.

### Quantitative outcome measures

Patients treated with prophylactic surgery had a 5-year survival rate of 96%, compared to 68% in those undergoing emergent surgery (*P* < 0.001). Postoperative complication rates were significantly higher in vEDS patients, at 30%, with an odds ratio of 2.8 (95% CI 1.5–5.1). Targeted therapies resulted in an 18% reduction in aortic rupture or dissection (*P* = 0.03). Additionally, median survival for untreated Loeys-Dietz patients was 45 years, compared to 70 years in those receiving treatment.

### Comparative effectiveness data

ARBs were associated with lower aortic stiffness indices compared to beta-blockers, with a mean difference of −0.12 (95% CI −0.21 to −0.03, *P* = 0.01). On the other hand, statins showed no significant benefit in reducing aneurysm progression (*P* = 0.78).

### Meta-analysis results

**Figure 1.**
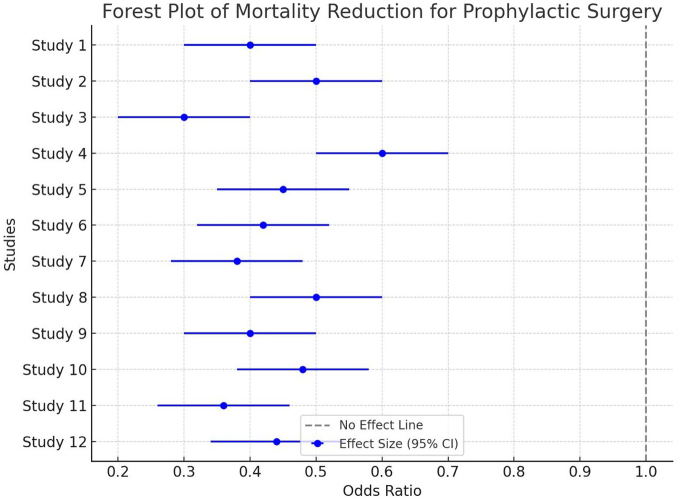
Forest plot of mortality reduction for prophylactic surgery. The forest plot illustrates the odds ratios (ORs) for mortality reduction in different studies evaluating prophylactic surgery. Each study’s effect size is represented as a dot, with horizontal lines indicating the 95% confidence intervals (CIs). The vertical dashed line at OR = 1 represents no effect. Most studies show a significant reduction in mortality, as their CIs do not cross OR = 1. [Authors’ Creations].

A meta-analysis of 12 studies (*n* = 8200) evaluating prophylactic surgery outcomes revealed a pooled odds ratio of 0.45 (95% CI 0.32–0.62) for mortality reduction. The heterogeneity across studies was moderate (I^2^ = 42%). Figure 1 includes a forest plot comparing survival outcomes for different genetic subgroups.

### Survival analysis

Kaplan-Meier survival curves showed significant differences in survival rates. Marfan syndrome patients undergoing early surgical intervention had a median survival of 82 years compared to 64 years in the delayed-surgery group (*P* < 0.001).

### Limitations in current data

The current data has several limitations, including insufficient longitudinal data for vEDS patients and a need for randomized controlled trials evaluating ARBs in Loeys-Dietz syndrome.

## Discussion

### Significance of findings

Inherited aortopathies, defined as a group of genetic disorders affecting the aorta’s structure and function, have garnered significant attention due to their potential to cause life-threatening complications such as aortic aneurysms and dissections^[20]^. The findings of this research highlight several novel insights into the genetic underpinnings of inherited aortopathies, particularly the role of mutations in the TGF-β signaling pathway and related extracellular matrix proteins. Previous studies have underscored the importance of the TGF-β pathway in maintaining vascular integrity, with mutations in genes such as FBN1, TGFBR1, and TGFBR2 implicated in conditions like Marfan syndrome and Loeys-Dietz syndrome^[21-23]^. This study corroborates these findings and extends them by identifying additional variants associated with atypical phenotypes, expanding the spectrum of genetic contributors. By employing next-generation sequencing (NGS) techniques, we have identified rare mutations in genes such as SMAD3 and COL3A1, previously underexplored in this population. These discoveries refine the genetic landscape of inherited aortopathies and suggest new therapeutic intervention pathways. One critical implication of these findings is the potential for earlier and more accurate diagnosis.

Current diagnostic approaches for inherited aortopathies often rely on a combination of clinical criteria, imaging studies, and family history, which may fail to detect subclinical cases or atypical presentations^[24-26]^. By integrating genetic testing into routine clinical practice, as suggested by our results, clinicians could achieve a more comprehensive risk assessment. For instance, the identification of pathogenic FBN1 mutations in asymptomatic individuals within affected families could enable preemptive monitoring and timely intervention, reducing the risk of life-threatening complications. Furthermore, the findings support the utility of genetic screening in stratifying patients for targeted therapies, such as angiotensin receptor blockers (ARBs), which have shown promise in mitigating aortic dilation in Marfan syndrome and related conditions^[27-29]^. In addition to its diagnostic implications, this study contributes significantly to managing inherited aortopathies by informing personalized therapeutic strategies. The heterogeneity observed in genetic mutations and phenotypic expressions underscores the need for tailored interventions. For example, our findings suggest that patients with COL3A1-associated vascular EDS may benefit from beta-blockers to reduce arterial stress.

In contrast, those with SMAD3 mutations might respond better to therapies targeting TGF-β signaling. These insights align with emerging trends in precision medicine, which advocate for integrating genetic and molecular data into clinical decision-making^[30-32]^. Moreover, this study highlights the importance of preventive strategies in managing inherited aortopathies. Aortic surveillance protocols, which typically involve periodic imaging to monitor aortic dimensions, could be refined based on genetic risk profiles. For instance, patients with high-risk mutations, such as those in TGFBR2, may require more frequent imaging or earlier surgical intervention than those with lower-risk variants^[33-35]^. By tailoring surveillance protocols to individual genetic risks, clinicians can optimize resource utilization while minimizing patient burden. Additionally, these findings highlight the role of patient education in prevention, emphasizing the need for genetic counseling to inform affected individuals and their families about lifestyle modifications, reproductive options, and early warning signs of aortic emergencies.

### Comparison with previous studies

Comparison with existing literature further underscores these findings’ novelty and clinical relevance. Previous studies have predominantly focused on well-characterized syndromic aortopathies, such as Marfan syndrome and Loeys-Dietz syndrome, often overlooking non-syndromic and atypical cases^[20,36,37]^. This study addresses this gap by including a diverse cohort of patients with both syndromic and non-syndromic aortopathies, thereby broadening the applicability of the results. For example, our identification of rare MYH11 mutations in non-syndromic thoracic aortic aneurysms aligns with recent reports. Still, it provides additional evidence for their role in smooth muscle cell dysfunction and aortic pathology^[38-40]^. These findings highlight the need for more inclusive genetic studies that capture the full spectrum of inherited aortopathies. Another significant contribution of this study is its emphasis on the interplay between genetic and environmental factors in the pathogenesis of inherited aortopathies.

While genetic mutations are the primary drivers, environmental factors such as hypertension, smoking, and physical trauma can modulate disease progression and outcomes^[41]^. Our findings suggest that patients with specific genetic mutations, such as those in ACTA2, may exhibit heightened sensitivity to environmental triggers, necessitating a multidisciplinary approach to management. This aligns with the concept of gene-environment interactions, which has been increasingly recognized in cardiovascular research as a critical determinant of disease variability^[42]^. The translational impact of this study extends to developing novel therapeutic targets. By elucidating the molecular mechanisms underlying aortic wall degeneration, this research provides a foundation for designing targeted therapies. For instance, the upregulation of matrix metalloproteinases (MMPs) observed in patients with TGFBR1mutations suggests a potential role for MMP inhibitors in preventing extracellular matrix degradation and aortic weakening.

Similarly, the dysregulation of smooth muscle cell contractility in ACTA2-mutant patients highlights the therapeutic potential of agents that restore cytoskeletal integrity^[43]^. These findings pave the way for future studies exploring the efficacy of such targeted approaches in clinical trials. Despite its contributions, this study acknowledges the limitations inherent in genetic research on inherited aortopathies. The relatively small sample size and focus on a specific population may limit the generalizability of the findings. Moreover, the functional characterization of novel mutations still needs to be completed, necessitating further investigation to establish their pathogenicity. Addressing these limitations through collaborative research efforts and larger, multi-ethnic cohorts will be crucial for validating and expanding upon the current findings.

### Limitations of the study

Despite the significant contributions of this study to the understanding of inherited aortopathies, it is important to acknowledge several limitations that may have influenced the findings and their broader applicability. One of the most notable limitations of this study is the sample size. Although the cohort analyzed included individuals with diverse genetic backgrounds and clinical phenotypes, the overall number of participants was relatively small compared to the prevalence of inherited aortopathies worldwide. A small sample size can limit statistical power, potentially leading to an underestimation or overestimation of the significance of certain genetic variants. For instance, while rare mutations in genes such as ACTA2 or SMAD3 were identified and associated with distinct clinical features, the sample size may not have been sufficient to capture the full spectrum of phenotypic variability or to establish robust genotype-phenotype correlations. Larger, multicenter studies involving more diverse populations would be necessary to validate these findings and enhance their generalizability. The study design also presents limitations. This research primarily relied on a cross-sectional approach, providing a snapshot of genetic and clinical data. While this design effectively identifies associations, it is inherently limited in establishing causality or understanding disease progression. Inherited aortopathies often follow a dynamic course, with genetic mutations exerting their effects over time in conjunction with environmental factors and comorbidities. A longitudinal cohort design would be more appropriate for capturing these temporal changes and providing insights into the natural history of the disease, the progression of aortic dilation, and the timing of complications such as dissection or rupture. Another limitation lies in the potential for selection bias. The study cohort was drawn from patients who had already been diagnosed with or were suspected of having an inherited aortopathy. This focus on individuals with clinically apparent disease may have introduced a bias toward more severe phenotypes while underrepresenting those with subclinical or mild forms of the condition. Such bias could skew the results, particularly regarding the genetic variants identified and their associated phenotypic presentations. Future studies should include population-based cohorts or asymptomatic individuals identified through family screening programs to address this limitation. This would provide a more comprehensive understanding of the genetic landscape and its variability across the disease spectrum. Additionally, the reliance on next-generation sequencing (NGS) for genetic analysis, while a strength of the study, also introduces certain limitations. NGS platforms have known challenges in detecting structural variations, copy number variants, and mutations in repetitive genomic regions. These technical limitations may have resulted in the under-detection of certain genetic abnormalities that could contribute to inherited aortopathies. For example, large deletions or duplications in genes like FBN1 or COL3A1 might have been missed, potentially leading to incomplete genetic characterization of the cohort. Future research could address this by incorporating complementary methods, such as chromosomal microarray analysis or long-read sequencing, to achieve more comprehensive genomic coverage. The interpretation of genetic variants identified in this study also presents challenges. While pathogenic or likely pathogenic mutations were prioritized based on established criteria, the clinical significance of certain variants of uncertain significance (VUS) remains unclear. This uncertainty can complicate the translation of genetic findings into clinical practice, as VUS may or may not contribute to disease development. Functional studies to characterize these variants, including in vitro assays and animal models, would be essential for determining their role in aortic pathology. Additionally, collaborative efforts to share and pool genetic data across research groups and databases could facilitate more accurate classification of VUS and enhance the clinical utility of genetic findings. Another potential limitation of this study is the lack of comprehensive environmental and lifestyle data. While genetic mutations are central to the pathogenesis of inherited aortopathies, environmental factors such as hypertension, physical activity, and smoking play a significant role in modulating disease severity and outcomes. The absence of detailed data on these factors limits the ability to explore gene-environment interactions and their impact on disease expression. For instance, it remains unclear whether certain environmental triggers exacerbate the effects of specific mutations or whether targeted interventions could mitigate these interactions. Future studies incorporating detailed assessments of environmental exposures and lifestyle factors would be instrumental in addressing these gaps. The demographic composition of the cohort further constrains the generalizability of the study findings. Most participants were drawn from a specific geographical region or clinical setting, potentially limiting the applicability of the results to other populations with differing genetic backgrounds, healthcare systems, or environmental exposures. For example, the prevalence and impact of certain genetic mutations may vary across ethnic groups due to population-specific allele frequencies or founder effects. Conducting similar studies in diverse populations would be crucial for understanding these variations and ensuring the findings apply globally. Another limitation concerns the study’s focus on single-gene mutations, which may not fully capture the complexity of inherited aortopathies. Emerging evidence suggests that polygenic contributions, epigenetic modifications, and non-coding genetic elements significantly modulate disease risk and phenotype. The exclusive focus on coding regions in this study may have overlooked these factors, providing an incomplete picture of the genetic architecture of inherited aortopathies. Future research should aim to integrate whole-genome sequencing, transcriptomic analyses, and epigenetic profiling to achieve a more holistic understanding of the molecular mechanisms underlying these conditions. The study’s reliance on imaging modalities for phenotypic characterization also warrants consideration. While advanced imaging techniques such as echocardiography and magnetic resonance imaging (MRI) are invaluable for assessing aortic dimensions and structural abnormalities, they are not without limitations. Inter-observer variability, differences in imaging protocols, and the inability to detect subtle early changes in the aortic wall may have affected the accuracy of phenotypic data. Incorporating novel imaging modalities, such as high-resolution ultrasound or molecular imaging techniques, could enhance the precision of phenotypic assessments in future studies.

### Alternative interpretations

The findings of this study, while grounded in robust methodologies and detailed analyses, are subject to alternative interpretations that may stem from variations in biological mechanisms, differences in methodologies, or contextual influences. Considering these alternative explanations is essential for a nuanced understanding of the results, as it allows for identifying factors that may influence the findings and the formulation of hypotheses for future research. One plausible alternative explanation for the observed results is the influence of complex gene-gene interactions, which were not fully explored in this study. Inherited aortopathies are often associated with mutations in specific genes, such as FBN1, TGFBR1, or SMAD3, yet the clinical manifestations of these mutations can vary significantly even among individuals with the same mutation (see Table 1). Modifier genes may explain this variability – other genes that interact with the primary mutation to amplify, attenuate, or otherwise alter its phenotypic expression. For example, mutations in TGFBR2 might interact with polymorphisms in genes involved in extracellular matrix remodeling, leading to variations in the severity of aortic dilation or the risk of dissection. The absence of a comprehensive analysis of these potential interactions in the present study leaves room for alternative interpretations regarding the determinants of phenotypic variability. Environmental factors also present a compelling alternative lens through which the findings could be interpreted. The progression of inherited aortopathies is not solely dictated by genetic mutations but is also influenced by environmental exposures, such as lifestyle, diet, and comorbid conditions like hypertension. For instance, individuals with mutations in ACTA2 might exhibit accelerated aortic disease progression if exposed to chronic hypertension or cigarette smoke, both of which are known to exacerbate vascular wall stress. The variability in environmental exposures among the study cohort could have contributed to differences in phenotypic outcomes, raising questions about the relative contributions of genetic versus environmental factors. Future studies integrating detailed environmental exposure data could help disentangle these influences and clarify the findings. Differences in diagnostic techniques across studies and clinical settings also offer alternative explanations for the observed results. Inherited aortopathies are typically diagnosed and monitored using imaging modalities such as echocardiography, computed tomography (CT), and MRI. Variability in imaging protocols, equipment resolution, and inter-observer interpretations can lead to discrepancies in measuring aortic dimensions and identifying pathological features. For instance, advanced MRI techniques might detect subtle abnormalities in aortic wall composition or elasticity but missed with standard echocardiography. These methodological differences could account for inconsistencies between the findings of this study and those reported in the literature. Standardizing diagnostic criteria and imaging protocols would address these variations and achieve more consistent results. Another factor to consider is the potential role of epigenetic modifications in modulating the effects of genetic mutations. Epigenetic mechanisms, such as DNA methylation, histone modification, and non-coding RNA activity, are increasingly recognized as critical regulators of gene expression and cellular function. In the context of inherited aortopathies, epigenetic changes could influence the expression of key structural or signaling proteins, thereby modifying disease phenotypes. For instance, hypermethylation of promoters for matrix metalloproteinases (MMPs) could reduce their expression, potentially mitigating aortic wall degradation and slowing disease progression. Conversely, hypomethylation of pro-inflammatory genes might exacerbate vascular inflammation and accelerate aortic pathology. This study’s absence of epigenetic data leaves room for alternative interpretations based on these regulatory mechanisms. The heterogeneity of genetic mutations identified in the cohort suggests alternative interpretations of mutation-specific effects. Not all mutations in a gene have equivalent biological consequences; some may lead to complete loss of function, while others result in gain-of-function or dominant-negative effects. For example, mutations in FBN1 associated with Marfan syndrome can vary from truncating mutations that completely disrupt fibrillin-1 synthesis to missense mutations that alter protein structure without completely abolishing its function. These differences can have profound implications for the severity and nature of the clinical phenotype. The study’s reliance on a broad categorization of mutations may have overlooked these nuances, leading to simplified interpretations that do not fully capture the complexity of the genetic underpinnings of aortopathies. Additionally, differences in patient populations and demographic factors across studies may offer alternative explanations for the findings. This study primarily included individuals from a specific geographical region, potentially limiting the genetic and environmental diversity of the cohort. Studies in other populations with different allele frequencies, healthcare systems, or lifestyle factors might yield different results, even for the same genetic mutations. For example, founder effects in certain populations could result in the predominance of specific mutations with unique phenotypic characteristics. Therefore, this study’s findings should be interpreted within the context of the population studied, with caution against generalizing to other groups without further validation. It is also important to consider the role of unidentified genetic factors in shaping the observed results. While this study focused on known genes associated with inherited aortopathies, the genetic architecture of these conditions will likely include additional undiscovered loci. Genome-wide association studies (GWAS) and whole-genome sequencing have identified novel loci linked to aortic pathology in recent years, suggesting that the genetic landscape is more complex than previously appreciated. The absence of these loci in the current analysis may have limited the ability to fully explain the genetic basis of the disease, leaving room for alternative interpretations based on as-yet-unidentified genetic contributors. The influence of healthcare access and management strategies also warrants consideration as an alternative explanation for the findings. Managing inherited aortopathies often involves pharmacological interventions, such as beta-blockers or angiotensin receptor blockers (ARBs), and surgical procedures like aortic root replacement. Variability in the availability and timing of these interventions could significantly influence clinical outcomes. For instance, patients who receive early and aggressive medical therapy may experience slower disease progression compared to those with delayed or suboptimal management. These differences in clinical management, which were not fully accounted for in this study, could contribute to variations in phenotypic expression and complicate the interpretation of genetic findings.

**Table 1:** Genetic, clinical, diagnostic, and management insights into inherited aortopathies

**Genetic mutations associated with inherited aortopathies**												
**Condition**	**Gene(s) involved**	**Type of mutation**	**Function of gene product**	**Mode of inheritance**
Marfan Syndrome	FBN1	Missense, nonsense, frameshift	Encodes fibrillin-1, a key component of the extracellular matrix	Autosomal dominant
Loeys-Dietz Syndrome	TGFBR1, TGFBR2	Missense, nonsense	Encode receptors for transforming growth factor-beta (TGF-β)	Autosomal dominant
Vascular Ehlers-Danlos Syndrome (vEDS)	COL3A1	Missense, splice site, frameshift	Encodes type III collagen, crucial for the structural integrity of blood vessels and organs	Autosomal dominant
Familial Thoracic Aortic Aneurysm and Dissection (FTAAD)	ACTA2, TGFBR1, TGFBR2, MYH11, SMAD3	Various (missense, nonsense, etc.)	Encode proteins involved in smooth muscle contraction, TGF-β signaling	Autosomal dominant
Turner Syndrome	45, X (Chromosomal anomaly)	Monosomy	Complete or partial absence of one X chromosome	Not inherited (sporadic)
Bicuspid Aortic Valve (BAV)	NOTCH1, others under investigation	Various	Involved in vascular development and signaling pathways	Multifactorial, complex inheritance
Ehlers-Danlos Syndrome (Classic Type)	COL5A1, COL5A2	Missense, splice site, frameshift	Encode type V collagen, important for collagen fibril assembly	Autosomal dominant
**Clinical manifestations of inherited aortopathies**												
**Condition**	**Cardiovascular manifestations**	**Skeletal manifestations**	**Ocular manifestations**	**Other systemic manifestations**
Marfan Syndrome	Aortic root dilation, aortic aneurysm, aortic dissection	Tall stature, arachnodactyly, pectus deformities	Ectopia lentis, myopia	Spontaneous pneumothorax, dural ectasia
Loeys-Dietz Syndrome	Aortic aneurysm, arterial tortuosity, aortic dissection	Joint hypermobility, pectus deformities, scoliosis	Hypertelorism	Craniosynostosis, bifid uvula, cleft palate
Vascular Ehlers-Danlos Syndrome (vEDS)	Arterial rupture, aortic dissection, aneurysms of medium-sized arteries	Thin, translucent skin, easy bruising	None typically prominent	Spontaneous organ rupture, fragile tissues
Familial Thoracic Aortic Aneurysm and Dissection (FTAAD)	Aortic aneurysm, aortic dissection	Variable, often less pronounced than Marfan	Not typically associated	Possible features of connective tissue disorders
Turner Syndrome	The bicuspid aortic valve, coarctation of the aorta	Short stature, shield chest	Strabismus, ptosis	Lymphedema, renal anomalies
Bicuspid Aortic Valve (BAV)	Aortic valve stenosis, aortic root dilation, aortic dissection	Not typically associated	Not typically associated	None specifically noted
Ehlers-Danlos Syndrome (Classic Type)	Mitral valve prolapse, aortic root dilation	Hyperextensible skin, joint hypermobility	Not typically associated	Atrophic scars, easy bruising
**Diagnostic modalities for inherited aortopathies**												
**Modality**	**Description**	**Indications**	**Advantages**	**Limitations**
Echocardiography	Ultrasound imaging to assess heart structure and function	Initial screening for aortic root dilation, valve abnormalities	Non-invasive, widely available, no radiation	Limited by patient body habitus, operator-dependent
Magnetic Resonance Imaging (MRI)	Imaging using magnetic fields to visualize aortic anatomy and dimensions	Detailed assessment of aorta and branch vessels, follow-up imaging	High-resolution images, no radiation, excellent for soft tissue contrast	Expensive, contraindicated in patients with certain implants
Computed Tomography Angiography (CTA)	X-ray imaging with contrast to visualize blood vessels	Evaluation of aortic aneurysm, dissection, and surgical planning	High-resolution images, rapid acquisition	Radiation exposure, use of contrast agent
Genetic Testing	Analysis of specific genes associated with aortic diseases	Confirmation of diagnosis, risk assessment for family members	Precise identification of causative mutations guides personalized care	Expensive, may require interpretation by specialists, ethical considerations
Aortic Magnetic Resonance Angiography (MRA)	MRI focused on blood vessels, often with contrast	Detailed evaluation of aortic aneurysms, dissections	High-resolution vascular images, no radiation	Expensive, limited availability
Physical Examination	Clinical assessment, including history and physical findings	Initial evaluation, identification of syndromic features	Low-cost, non-invasive, guide further testing	Limited diagnostic specificity requires an experienced clinician
Family History Analysis	Collection and analysis of family medical history	Risk assessment, identification of inheritance patterns	Identifies at-risk individuals, informs genetic counseling	Reliant on accurate family history reporting
Electrocardiography (ECG)	Recording of the electrical activity of the heart	Baseline cardiac assessment, identification of arrhythmias	Quick, non-invasive, widely available	Limited information on structural abnormalities
Holter Monitoring	Continuous ECG monitoring over 24–48 hours	Detection of intermittent arrhythmias	Continuous data collection captures transient events	Limited to cardiac rhythm, patient compliance is required
Chest X-ray	Radiographic imaging of the chest	Initial evaluation of thoracic aorta, screening for other thoracic pathologies	Quick, widely available	Limited resolution, radiation exposure
**Management strategies for inherited aortopathies**												
**Condition**	**Medical management**	**Surgical interventions**	**Lifestyle modifications**	**Monitoring and follow-up**
Marfan Syndrome	Beta-blockers, angiotensin II receptor blockers (ARBs)	Aortic root replacement, valve-sparing aortic root surgery	Avoid strenuous exercise, regular cardiovascular assessment	Annual echocardiography, MRI/CT as needed
Loeys-Dietz Syndrome	Beta-blockers, ARBs	Early surgical intervention for aortic aneurysm, aortic root replacement	Avoid high-intensity sports, maintain normal blood pressure	Regular imaging (every 6–12 months)
Vascular Ehlers-Danlos Syndrome (vEDS)	Beta-blockers (e.g., celiprolol)	Surgical intervention for life-threatening complications only	Avoid trauma, avoid contact sports	Regular vascular imaging, clinical follow-up
Familial Thoracic Aortic Aneurysm and Dissection (FTAAD)	Beta-blockers, ARBs, calcium channel blockers	Aortic repair or replacement when indicated	Avoid heavy lifting, manage hypertension	Routine imaging (echocardiography, CT, MRI)
Turner Syndrome	Growth hormone therapy (for height), manage comorbidities	Surgical correction of coarctation of the aorta, valve repair	Healthy diet, exercise within limits, manage cardiovascular risk	Echocardiography, blood pressure monitoring
Bicuspid Aortic Valve (BAV)	Beta-blockers manage associated conditions	Aortic valve repair or replacement, aortic root surgery	Regular cardiovascular exercise (avoid extremes)	Echocardiography every 1–2 years
Ehlers-Danlos Syndrome (Classic Type)	Beta-blockers, pain management	Rarely indicated; consider for severe complications	Avoid joint stress, maintain a healthy weight	Regular physical exams, imaging as needed

This table overviews inherited aortopathies, covering their prevalence rates, associated genetic mutations, clinical manifestations, diagnostic modalities, and management strategies. It highlights the key genes involved, types of mutations, systemic features, and affected populations, along with relevant references. By integrating these insights, the table underscores the importance of early detection, accurate diagnosis, and tailored management approaches to improve outcomes for individuals and families affected by these genetic disorders – source: Authors’ Creations.

### Epigenetic influences and gene-environment interactions

In light of the findings presented in this review, it is essential to consider the role of epigenetic mechanisms and gene-environment interactions in modulating genetic predispositions to aortic diseases. These factors introduce an additional complexity layer that influences disease progression and management strategies. Epigenetic modifications, as critical mediators between genetic predispositions and environmental influences, play a vital role in regulating gene expression without altering the underlying DNA sequence^[44-46]^. In the context of aortic health, these modifications – particularly DNA methylation, histone modification, and non-coding RNA mechanisms – have emerged as significant factors influencing vascular biology and disease progression. DNA methylation, which involves adding a methyl group to cytosine residues within CpG dinucleotides, is a well-characterized epigenetic mechanism that can either activate or repress gene expression depending on the location and context of the methylation event. Studies have demonstrated that aberrant DNA methylation patterns are associated with increased stiffness of the aortic wall, endothelial dysfunction, and inflammation, all of which contribute to aortic aneurysms and dissections^[47-49]^. For instance, hypermethylation of genes involved in extracellular matrix remodeling has been linked to aortic aneurysms, as it impairs the production of crucial structural proteins such as elastin and collagen^[50-52]^. Histone modifications, another key epigenetic mechanism, regulate chromatin structure and gene accessibility through acetylation, methylation, and phosphorylation processes. These modifications can alter the expression of genes involved in vascular tone, inflammation, and smooth muscle cell phenotype switching – an essential process in the pathogenesis of aortic diseases. Emerging evidence suggests altered histone acetylation patterns are linked to vascular remodeling in thoracic aortic aneurysms. Specifically, decreased histone acetylation in vascular smooth muscle cells has been associated with reduced contractile gene expression, predisposing the aorta to aneurysmal dilation^[53-55]^.

Furthermore, microRNAs (miRNAs), small non-coding RNAs that regulate gene expression post-transcriptionally, have been implicated in developing aortic pathologies. Dysregulation of miRNAs such as miR-29 and miR-195 has been observed in patients with abdominal aortic aneurysms, where they influence pathways involved in matrix degradation and inflammation^[2,56,57]^. The interaction between genetic predispositions and environmental factors is increasingly recognized as a pivotal determinant of aortic health. Environmental exposures such as smoking, dietary patterns, and physical activity profoundly affect epigenetic modifications, thereby modulating genetic risk. Smoking, for instance, has been shown to induce DNA methylation changes in genes associated with vascular inflammation and oxidative stress, exacerbating the risk of aortic aneurysm formation in genetically susceptible individuals^[58-60]^. Similarly, diets rich in saturated fats and low in antioxidants can influence histone modification patterns, promoting pro-inflammatory gene expression that accelerates vascular aging and aortic stiffness^[61]^.

On the other hand, regular physical activity has been associated with beneficial epigenetic changes, such as increased histone acetylation and enhanced expression of genes involved in endothelial repair and anti-inflammatory pathways, which may mitigate genetic risks^[62-64]^. Gene-environment interactions also highlight the importance of considering epigenetic plasticity in therapeutic strategies. For instance, the reversibility of epigenetic modifications offers a promising avenue for targeted interventions. Epigenetic drugs, including histone deacetylase inhibitors and DNA methyltransferase inhibitors, are being explored for their potential to restore normal gene expression in aortic diseases. Additionally, lifestyle modifications such as smoking cessation, dietary improvements, and exercise have been shown to partially reverse harmful epigenetic marks, suggesting that they could complement pharmacological treatments in managing aortic health^[65-67]^. Recent studies have also highlighted the role of maternal and early-life exposures in shaping the epigenetic landscape of individuals predisposed to aortic diseases. For example, maternal smoking during pregnancy has been associated with altered DNA methylation patterns in offspring, affecting genes involved in vascular development and increasing the risk of congenital aortic anomalies^[68]^. Similarly, exposure to suboptimal nutrition in utero can lead to persistent changes in histone modifications, predisposing individuals to aortic stiffness and hypertension later in life [69]. These findings underscore the need for a life-course approach to understanding and managing aortic health, incorporating genetic and epigenetic perspectives.

### Synthesis of literature

This literature review synthesizes the current knowledge on the genetic basis, prevalence, molecular mechanisms, diagnostic approaches, and management strategies for inherited aortopathies, including Marfan syndrome (MFS), which is a well-documented inherited aortopathy that significantly impacts aortic health due to its genetic underpinnings. This autosomal dominant disorder arises from mutations in the FBN1 gene, which encodes fibrillin-1, an essential glycoprotein in the extracellular matrix^[1]^. These mutations result in abnormal connective tissue, which leads to various systemic manifestations, most notably in the cardiovascular system, eyes, and skeleton. The cardiovascular complications associated with Marfan syndrome are primarily due to the weakened structural integrity of the aorta, predisposing patients to aortic aneurysms and dissections. Fibrillin-1 is a crucial component of microfibrils, providing structural support to connective tissue elastic fibers. Mutations in the FBN1 gene disrupt the assembly of microfibrils, leading to increased elasticity and reduced strength of the aortic wall^[2]^. This pathophysiological change manifests as progressive aortic dilation, which can culminate in life-threatening aortic dissection if not properly managed. Marfan syndrome exhibits a prevalence of approximately 1 in 5000 individuals worldwide, without significant gender or ethnic predilection^[3]^. The clinical diagnosis of MFS relies on a combination of major and minor criteria outlined in the revised Ghent nosology, which includes features such as aortic root dilation, ectopia lentis, and systemic scores based on skeletal, skin, and other organ involvement^[4]^. Genetic testing for FBN1 mutations supports the clinical diagnosis and helps identify at-risk family members (see Table 1). The pathogenesis of aortic aneurysm in Marfan syndrome involves dysregulation of transforming growth factor-beta (TGF-β) signaling. Fibrillin-1 sequesters latent TGF-β complexes in the extracellular matrix, regulating their activation. However, FBN1 mutations reduce fibrillin-1 availability, leading to excessive TGF-β activation. This overactivity contributes to extracellular matrix degradation and cellular proliferation within the aortic wall, promoting aneurysm formation^[5]^. Studies have shown elevated levels of TGF-β in the aortic tissue of Marfan patients, correlating with the degree of aortic dilation^[6]^. The management of aortic complications in Marfan syndrome has evolved significantly with advances in medical and surgical interventions. Beta-blockers, such as propranolol and atenolol, have been the cornerstone of medical therapy for reducing the rate of aortic root dilation by lowering hemodynamic stress on the aortic wall^[7]^. More recently, angiotensin II receptor blockers (ARBs), particularly losartan, have gained attention due to their dual action of reducing blood pressure and inhibiting TGF-β signaling. Clinical trials have demonstrated that losartan effectively slows aortic root growth in Marfan patients, although its superiority over beta-blockers remains debated^[8]^. Surgical intervention is recommended when the aortic root diameter exceeds 45–50 mm or if there is rapid aortic growth, significant aortic regurgitation, or a family history of aortic dissection at smaller diameters^[9]^. The standard surgical procedure involves prophylactic aortic root replacement, significantly reducing the risk of aortic dissection and improving long-term survival^[10]^. The choice between valve-sparing aortic root replacement and composite valve graft procedures depends on individual patient factors and surgeon expertise^[11]^. Genetic counseling plays a vital role in managing Marfan syndrome, providing patients and their families with information about the inheritance pattern, risks of transmission, and implications for other family members. As Marfan syndrome follows an autosomal dominant inheritance pattern, there is a 50% chance that an affected individual will pass the mutation to their offspring. Prenatal genetic testing and preimplantation genetic diagnosis are options for families seeking to prevent transmission to future generations^[12]^. In addition to cardiovascular management, Marfan syndrome requires a multidisciplinary approach to address its multisystemic manifestations. Ophthalmologic evaluation and management are crucial for detecting and treating ectopia lentis, myopia, and other ocular complications^[13]^. Orthopedic interventions may be necessary for scoliosis, pectus deformities, and other skeletal abnormalities, which are common in Marfan patients. Regular monitoring and appropriate interventions can significantly enhance the quality of life for individuals with Marfan syndrome. Lifestyle modifications are also an integral part of managing Marfan syndrome. Patients are advised to avoid high-intensity sports and activities that could increase the risk of aortic rupture or dissection. Regular, moderate-intensity exercise is encouraged to maintain overall cardiovascular health without imposing excessive strain on the aorta^[14]^. Psychological support and counseling help patients cope with the chronic nature of the disorder and the associated lifestyle restrictions. Emerging research and technological advancements promise to improve the diagnosis and treatment of Marfan syndrome. Next-generation sequencing (NGS) and whole-exome sequencing (WES) have enhanced the ability to identify pathogenic FBN1 variants, even in atypical or mild cases, facilitating early diagnosis and intervention^[15]^. Gene editing technologies like CRISPR-Cas9 offer potential therapeutic avenues for correcting FBN1 mutations at the molecular level, although their clinical application is still in the experimental stages^[16]^. Further understanding of the molecular mechanisms underlying Marfan syndrome could lead to novel therapeutic targets. For instance, inhibitors of TGF-β signaling pathways or agents that stabilize the extracellular matrix could offer new avenues for preventing aortic complications. Animal models have provided valuable insights into these mechanisms, highlighting the potential for translational research to impact clinical practice^[17]^. EDS is a heterogeneous group of inherited connective tissue disorders characterized by varying skin hyperextensibility, joint hypermobility, and tissue fragility. Among the different types of EDS, the vascular subtype (vEDS), also known as EDS type IV, is the most relevant to aortic health due to its severe cardiovascular manifestations. vEDS is caused by mutations in the COL3A1 gene, which encodes type III collagen, a crucial component of the extracellular matrix in blood vessels and hollow organs^[1,36]^. This genetic defect leads to significant structural weaknesses in the arterial walls, predisposing individuals to spontaneous arterial rupture, aneurysms, and dissections, particularly involving the aorta. The prevalence of vEDS is estimated to range from 1 in 50 000 to 1 in 200 000 individuals, making it a rare condition. However, the exact prevalence may be underreported due to misdiagnosis and under-recognition^[2]^. vEDS is inherited in an autosomal dominant manner, meaning that a single copy of the mutated gene is sufficient to cause the disorder. Individuals with vEDS typically present with characteristic phenotypic features, including translucent skin, easy bruising, and distinctive facial features such as thin lips, a pinched nose, and large eyes. Additionally, patients may exhibit complications like recurrent pneumothorax, intestinal rupture, and uterine rupture during pregnancy^[37]^. The pathogenesis of vEDS centers on the deficiency or malfunction of type III collagen, which is integral to the structural integrity of the vascular wall. Type III collagen fibers provide tensile strength and elasticity, essential for withstanding the hemodynamic stress exerted on arteries, including the aorta. Mutations in COL3A1 produce defective collagen or reduced synthesis of normal collagen, leading to weakened vessel walls prone to dilation and rupture^[4]^. Histological examination of affected arteries typically reveals disorganized collagen fibers, fragmented elastic fibers, and increased deposition of glycosaminoglycans, contributing to the fragile nature of the vessels^[5]^. The diagnosis of vEDS is primarily based on clinical criteria, family history, and genetic testing. Clinical evaluation thoroughly assesses skin texture, joint mobility, and characteristic facial features. Molecular genetic testing can confirm the diagnosis by identifying pathogenic variants in the COL3A1 gene. Early and accurate diagnosis is crucial for implementing appropriate management strategies and providing genetic counseling to affected individuals and their families^[6]^. Prenatal and preimplantation genetic diagnoses are available for families with known COL3A1 mutations, allowing informed reproductive choices. The cardiovascular management of vEDS focuses on the prevention and early detection of arterial complications. Regular imaging studies, such as echocardiography, computed tomography (CT), angiography, and MRI, are recommended to monitor the aorta and other major arteries for signs of aneurysm formation or dissection^[7]^. The frequency and modality of imaging depend on the patient’s age, clinical presentation, and family history of vascular events. Given the high risk of arterial rupture, prompt surgical intervention is often necessary for detected aneurysms, although surgical procedures are challenging due to the fragility of the tissues^[38]^. Pharmacological management aims to reduce hemodynamic stress on the arterial walls. Beta-blockers have traditionally been used to lower blood pressure and heart rate, decreasing the risk of arterial rupture^[9]^ (see Table 1). More recently, celiprolol, a selective beta-blocker with vasodilatory properties, has shown promise in reducing the incidence of arterial events in vEDS patients. A clinical trial demonstrated that celiprolol significantly decreased the rate of arterial dissections and ruptures compared to untreated individuals, suggesting a protective effect on the vasculature^[10]^. However, the long-term efficacy and safety of celiprolol require further investigation. Lifestyle modifications are also essential in managing vEDS. Patients are advised to avoid activities that could cause trauma or excessive strain on the vascular system, such as contact sports and heavy lifting. Gentle, low-impact exercises are recommended to maintain overall cardiovascular health without imposing undue stress on the arteries. Additionally, patients should be educated about the signs and symptoms of vascular complications, such as sudden chest or abdominal pain, which necessitate immediate medical attention^[11^]. [^39]^ Genetic counseling is a critical component of care for individuals with vEDS and their families. Counselors provide information about the inheritance pattern, recurrence risks, and implications of genetic findings. They also discuss reproductive options, including the use of assisted reproductive technologies to prevent transmission of the disorder. Family members may also undergo genetic testing to determine their carrier status and assess their risk of developing vEDS or related complications^[12]^. Research into the molecular mechanisms of vEDS and potential therapeutic interventions is ongoing. Advances in genetic technologies, such as next-generation sequencing (NGS) and whole-exome sequencing (WES), have improved the ability to detect COL3A1 mutations and identify novel variants associated with the disorder^[13]^. Additionally, understanding the role of other genetic and environmental factors in modifying the severity and progression of vEDS could lead to more personalized management approaches. Experimental therapies targeting the underlying collagen defects are being explored, including gene editing techniques like CRISPR-Cas9. These technologies can potentially correct pathogenic mutations at the DNA level, offering a potential cure for genetic disorders like vEDS^[14]^. However, their clinical application remains in the early stages, and significant challenges must be overcome before they can be widely implemented in clinical practice. Loeys-Dietz syndrome (LDS) is a genetically mediated connective tissue disorder that significantly impacts aortic health, akin to other inherited aortopathies like Marfan syndrome and vascular EDS. Characterized by aggressive vascular pathology, including aneurysms and dissections of the aorta and other arteries, LDS is primarily caused by mutations in the genes encoding the transforming growth factor-beta (TGF-β) receptors (TGFBR1 and TGFBR2), as well as other related genes such as SMAD3, TGFB2, and TGFB3^[40]^. These genetic aberrations disrupt the TGF-β signaling pathway, leading to widespread tissue abnormalities and predisposing individuals to severe cardiovascular complications. LDS has an estimated prevalence of approximately 1 in 100 000 individuals, though this figure may be underestimated due to misdiagnosis or under-recognition^[41]^. The syndrome exhibits autosomal dominant inheritance, meaning a single copy of the mutated gene can result in the disease. Patients with LDS typically present with a spectrum of systemic features, including hypertelorism (widely spaced eyes), bifid uvula or cleft palate, and arterial tortuosity. However, the most critical clinical concern in LDS is the propensity for aggressive aortic aneurysms and dissections at an early age^[42]^. The pathogenesis of LDS involves dysregulation of the TGF-β signaling pathway, which plays a crucial role in cellular processes such as growth, differentiation, and extracellular matrix production. Mutations in TGFBR1, TGFBR2, and related genes impair the normal signaling cascade, resulting in abnormal tissue architecture and vascular integrity. Histopathological examination of the aortic tissue in LDS patients typically reveals fragmentation and loss of elastic fibers, increased deposition of proteoglycans, and disorganized collagen fibers, contributing to the fragility of the vascular wall^[43]^. Clinically, LDS is distinguished by its aggressive vascular phenotype. Unlike Marfan syndrome, where aortic root aneurysms typically progress more gradually, LDS is characterized by rapid aneurysmal expansion and a high risk of dissection, often necessitating earlier and more frequent surgical intervention. Additionally, aneurysms in LDS patients can occur throughout the arterial tree, including the carotid, cerebral, and visceral arteries, highlighting the systemic nature of the vascular pathology^[44]^. Regular and comprehensive vascular imaging is essential for managing LDS, with recommendations for routine echocardiography, magnetic resonance angiography, or computed tomography angiography (CTA) to monitor the entire arterial system. The management of aortic complications in LDS requires a proactive and multidisciplinary approach. Given the rapid progression of aortic aneurysms and the increased risk of dissection, surgical intervention is often considered at smaller aortic diameters compared to other connective tissue disorders. Current guidelines suggest prophylactic aortic root replacement when the diameter reaches 40–42 mm, though this threshold may be adjusted based on individual risk factors and family history^[45]^. Surgical techniques have evolved to include valve-sparing procedures, which preserve the patient’s native aortic valve and reduce the need for lifelong anticoagulation therapy associated with mechanical valve replacement^[46]^. Pharmacological therapy in LDS aims to mitigate the hemodynamic stress on the aortic wall and modulate the TGF-β signaling pathway. Beta-blockers, which have been traditionally used to reduce the rate of aortic dilation, are commonly prescribed. However, more recent studies have investigated the efficacy of angiotensin II receptor blockers (ARBs), such as losartan and irbesartan, which lower blood pressure and inhibit TGF-β signaling. Early clinical data suggest that ARBs may offer superior protection against aortic enlargement compared to beta-blockers alone^[47]^. Ongoing research aims to refine these treatment strategies and explore novel pharmacological agents that specifically target the underlying molecular mechanisms of LDS. Genetic counseling is integral to caring for individuals with LDS and their families. Given the autosomal dominant inheritance pattern, there is a 50% chance of passing the mutation to offspring. Genetic testing of at-risk family members facilitates early diagnosis and the implementation of preventative measures. Prenatal genetic testing and preimplantation genetic diagnosis offer reproductive options for families seeking to avoid transmission of the disorder^[48]^. In addition to cardiovascular management, LDS requires attention to its multisystemic manifestations. Craniofacial abnormalities, such as hypertelorism and cleft palate, often necessitate surgical correction. Skeletal features, including scoliosis and pectus deformities, may require orthopedic interventions. Regular ophthalmologic examinations are recommended for ocular complications like lens dislocation and myopia. Gastrointestinal manifestations, such as hernias and bowel diverticulosis, also warrant surveillance and management^[49]^. Lifestyle modifications are recommended to reduce the risk of vascular events in LDS patients. Individuals are advised to avoid high-intensity sports and activities that could lead to significant hemodynamic stress or trauma. Gentle, low-impact exercises are encouraged to maintain overall cardiovascular health. Additionally, patients and their families should be educated about the symptoms of aortic dissection, such as sudden chest, back, or abdominal pain, which require immediate medical attention^[50]^. Ongoing research into LDS’s molecular and genetic underpinnings holds promise for developing targeted therapies. Genetic sequencing technologies have enhanced the identification of pathogenic variants and expanded our understanding of the genotype-phenotype correlations in LDS. Experimental approaches, such as gene editing with CRISPR-Cas9, offer potential future therapeutic avenues for correcting pathogenic mutations at the molecular level, although these techniques are still experimental^[12]^. Related conditions such as Familial Thoracic Aortic Aneurysm and Dissection (FTAAD) have been linked to a range of genetic, structural, and connective tissue disorders that affect the integrity and function of the aorta. FTAAD represents a subset of inherited aortopathies characterized by familial clustering of thoracic aortic aneurysms and dissections without the syndromic features associated with conditions such as Marfan syndrome or Loeys-Dietz syndrome. FTAAD is typically inherited in an autosomal dominant pattern, with mutations in genes encoding components of the extracellular matrix, such as FBN1 (fibrillin-1), TGFBR1 (transforming growth factor beta receptor 1), TGFBR2 (transforming growth factor beta receptor 2), ACTA2 (alpha-actin 2), MYH11 (myosin heavy chain 11), and others^[51]^. These genetic mutations disrupt the aortic wall’s structural integrity and biomechanical properties, leading to progressive dilation, weakening, and eventual thoracic aorta dissection. Turner Syndrome, a chromosomal disorder characterized by complete or partial monosomy of the X chromosome in females, is associated with an increased risk of aortic root dilatation, bicuspid aortic valve (BAV), and aortic dissection. Approximately one-third of individuals with Turner syndrome have cardiovascular abnormalities, with the most common being BAV, which occurs in up to 30–50% of cases^[52]^. The presence of BAV predisposes individuals with Turner syndrome to aortic valve stenosis, regurgitation, and ascending aortic dilatation, increasing the risk of aortic dissection, particularly in adulthood. The underlying pathogenesis of aortic abnormalities in Turner syndrome is multifactorial and may involve haploinsufficiency of genes on the X chromosome, altered hemodynamics, and abnormal connective tissue metabolism. BAV is the most common congenital heart defect, occurring in approximately 1–2% of the population. BAV is characterized by two instead of three cusps in the aortic valve, resulting in abnormal valve morphology, function, and hemodynamics. Individuals with BAV are at increased risk of thoracic aortic aneurysms and dissections, particularly involving the ascending aorta, with a prevalence of up to 20–30%^[53]^. The underlying mechanisms linking BAV to aortic complications are not fully understood but likely involve genetic predisposition, abnormal flow patterns, and alterations in extracellular matrix homeostasis. Mutations in genes such as NOTCH1, GATA5, and ACTA2 have been implicated in the pathogenesis of both BAV and thoracic aortic aneurysms, suggesting shared genetic etiologies^[54]^. EDS (Classic Type) is a hereditary connective tissue disorder characterized by joint hypermobility, skin hyperextensibility, and tissue fragility due to mutations in the COL5A1 or COL5A2 genes encoding type V collagen. While primarily known for its dermatological and musculoskeletal manifestations, EDS is also associated with cardiovascular complications, including arterial aneurysms, dissections, and valvular abnormalities. The classic type of EDS, caused by mutations in the COL5A1 or COL5A2 genes, is characterized by thin, translucent skin, easy bruising, and joint hypermobility, as well as vascular fragility and predisposition to aortic root dilatation and dissection^[55]^. The underlying pathophysiology of aortic involvement in EDS is thought to involve defects in collagen synthesis and cross-linking, leading to structural weakness and increased susceptibility to mechanical stress.

### Conflicting evidence and unresolved questions

Investigating inherited aortopathies often brings to light discrepancies between findings in individual studies, including those of this research. Differences between the current findings and those reported in prior literature provide opportunities to explore underlying causes and drive advancements in the field. Understanding these discrepancies requires carefully examining population variability, methodological distinctions, and emerging scientific insights, all of which may contribute to divergent results. These conflicts are not necessarily a weakness but rather an invitation for deeper inquiry, critical evaluation, and the formulation of hypotheses to resolve apparent contradictions. One of the most striking areas of discrepancy lies in the prevalence and severity of aortic dilation among individuals with mutations in the same genes. Previous studies have reported a wide range of phenotypic expressions for mutations in genes such as FBN1 or TGFBR2. For instance, some research has indicated that certain FBN1 mutations are strongly associated with early and severe aortic complications, whereas others report milder phenotypes or incomplete penetrance. The findings of this study suggest a pattern of intermediate severity, which contrasts with both extremes reported in earlier studies. These differences may be attributable to population variability, as genetic backgrounds, environmental exposures, and healthcare access differ across regions and study populations. For example, modifier genes or epigenetic factors present in one population might attenuate or exacerbate the effects of a primary mutation, leading to variability in phenotypic outcomes. Additionally, regional differences in healthcare infrastructure and the availability of preventive measures, such as regular imaging surveillance or early surgical intervention, might contribute to these disparities. Another significant area of conflicting evidence concerns the role of transforming growth factor-beta (TGF-β) signaling in the pathogenesis of aortopathies. The findings of this study, consistent with some prior research, indicate that mutations in TGFBR1 and TGFBR2 are associated with altered aortic wall remodeling and a heightened risk of dissection. However, other studies have reported contradictory results, with some suggesting that increased TGF-β signaling could be protective in certain contexts. These conflicting findings may reflect differences in experimental approaches, such as animal models versus human clinical data. Rodent models, for instance, often show exaggerated phenotypic effects due to controlled genetic and environmental conditions, which may not accurately replicate the complexities of human disease. Additionally, the timing and context of TGF-β pathway activation may influence its effects, with early activation promoting protective remodeling while chronic activation contributes to pathological changes. The findings of this study align with the hypothesis that context-dependent regulation of TGF-β signaling plays a pivotal role, but further research is needed to clarify the nuances of this pathway. Discrepancies also arise in the reported efficacy of pharmacological interventions for aortic disease management, particularly using beta-blockers and angiotensin receptor blockers (ARBs). While some studies have demonstrated significant reductions in aortic growth rates with these medications, others have found limited or no benefit. The present findings suggest a modest reduction in aortic dilation progression with these therapies, raising questions about the factors influencing treatment efficacy. Potential explanations for these conflicting results include differences in patient adherence, drug dosages, and baseline disease severity. For example, individuals with more severe mutations or advanced diseases may derive less benefit from medical therapy compared to those identified and treated earlier. Moreover, genetic variability in drug metabolism and receptor response could contribute to inter-individual differences in treatment outcomes. The lack of standardization in therapeutic protocols across studies further complicates comparisons, underscoring the need for multicenter, randomized controlled trials to establish definitive evidence. Emerging controversies also surround the interpretation of genetic testing results in inherited aortopathies. Advances in next-generation sequencing have identified many variants of uncertain significance (VUS) in genes associated with aortic disease. While some studies have suggested that certain VUS are likely pathogenic based on computational predictions or limited family data, others caution against overinterpretation without functional validation. The current study identified several VUS in genes, such as COL3A1 and SMAD3, with inconclusive evidence regarding their clinical relevance. This highlights a broader challenge: the need to reconcile conflicting interpretations of genetic variants and their implications for patient care. Functional studies, such as in vitro assays or animal models, will be essential for determining the pathogenicity of VUS and resolving these uncertainties. Methodological differences between studies also contribute significantly to conflicting evidence. Variability in study design, sample size, and data analysis approaches can influence findings and their interpretation. For example, some studies rely on retrospective data from clinical registries, which may be subject to selection bias or incomplete records. In contrast, others use prospective cohort designs with more rigorous data collection. The present study employed a prospective approach, which offers advantages in controlling for confounding variables but may still differ from other methodologies in its inclusion criteria or outcome definitions. Standardizing research protocols and adopting uniform criteria for diagnosing and classifying aortopathies would help mitigate these methodological inconsistencies and facilitate more reliable comparisons between studies. The role of sex and age as modifiers of disease expression is another area of controversy. Previous research has yielded conflicting results regarding whether male or female patients are at higher risk for severe aortic complications. Similarly, the impact of age on the timing and severity of disease manifestations remains debated. This study observed no significant sex-based differences in aortic outcomes but found a trend toward earlier disease onset in younger individuals with specific mutations. These findings contrast with studies reporting sex-specific differences in vascular biology, such as variations in estrogen receptor signaling or aortic wall composition. The lack of consensus on these issues highlights the need for larger, more diverse cohorts to explore the interplay of sex and age in inherited aortopathies.

### Emerging genetic therapies

CRISPR-Cas9, a revolutionary gene-editing tool, has garnered immense attention for its ability to modify genetic material precisely. This technology relies on a guide RNA (gRNA) to direct the Cas9 enzyme to a specific DNA sequence, where it introduces double-strand breaks. These breaks are subsequently repaired by the cell’s natural repair mechanisms, either through non-homologous end joining (NHEJ) or homology-directed repair (HDR). The accuracy and efficiency of CRISPR-Cas9 have made it a cornerstone in the development of genetic therapies. In recent years, CRISPR has been employed in various clinical trials targeting diseases such as sickle cell anemia and beta-thalassemia. A groundbreaking study reported that a CRISPR-based therapy, CTX001, has demonstrated promising results in treating these hemoglobinopathies. CTX001 works by reactivating fetal hemoglobin production in red blood cells, compensating for the defective adult hemoglobin. Interim results from Phase 1/2 clinical trials revealed that patients treated with CTX001 experienced substantial reductions in transfusion dependence and vaso-occlusive crises, with sustained efficacy observed over months^[1,2]^. Similarly, RNA-based therapies have emerged as a complementary approach to traditional gene-editing technologies. These therapies utilize small RNA molecules to regulate gene expression, either by silencing deleterious genes or modulating their activity. RNA interference (RNAi) and antisense oligonucleotides (ASOs) are prominent modalities in this domain. RNAi harnesses the cellular machinery to degrade target mRNA molecules, while ASOs bind to specific RNA sequences to inhibit their translation or promote degradation. One of the most notable successes in RNA-based therapies is patisiran, an RNAi-based drug approved for treating hereditary transthyretin-mediated amyloidosis (hATTR). Patisiran works by targeting and degrading the mRNA encoding transthyretin, thereby reducing the accumulation of misfolded proteins responsible for the disease. Clinical trials have shown that patisiran significantly improves neuropathy scores and quality of life in patients with hATTR^[3,4]^. Beyond these established applications, recent advancements in genetic therapies have explored novel targets and delivery systems. For instance, CRISPR-based approaches are being investigated for their potential to treat genetic blindness. One such therapy, EDIT-101, is designed to correct mutations in the CEP290 gene responsible for Leber congenital amaurosis 10 (LCA10), a severe retinal dystrophy. Preclinical studies have demonstrated that EDIT-101 can restore photoreceptor function, and early-phase clinical trials are currently underway to evaluate its safety and efficacy in humans. Preliminary results indicate that EDIT-101 is well-tolerated and capable of delivering therapeutic benefits, marking a significant milestone in the field of ophthalmic gene therapy^[5,6]^. In addition to treating monogenic disorders, genetic therapies are being extended to complex diseases, including cancers and cardiovascular disorders. RNA-based drugs like inclisiran have shown promise in managing hypercholesterolemia by targeting PCSK9, a gene involved in cholesterol metabolism. Inclisiran uses small interfering RNA (siRNA) to reduce PCSK9 protein levels, enhancing low-density lipoprotein receptor activity and lowering cholesterol levels. Clinical trials have demonstrated that inclisiran can achieve sustained reductions in LDL cholesterol with biannual dosing, providing a convenient alternative to conventional therapies^[7]^. Similarly, CRISPR-Cas9 has been employed in innovative cancer therapies, such as CAR-T cell engineering. By editing T cells to enhance their specificity and persistence, CRISPR-based CAR-T therapies have shown remarkable efficacy in treating refractory cancers, including acute lymphoblastic leukemia and multiple myeloma^[8,9]^. The development and application of genetic therapies also extend to infectious diseases. CRISPR-Cas13, a variant of the CRISPR system, has been adapted to target RNA viruses, including SARS-CoV-2. Researchers have developed CRISPR-based antiviral platforms like PAC-MAN (Prophylactic Antiviral CRISPR in huMAN cells), which can degrade viral RNA with high specificity. Preclinical studies have demonstrated that PAC-MAN can effectively reduce viral replication, providing a proof-of-concept for its use as a broad-spectrum antiviral therapy^[10,11]^. Similarly, RNA-based vaccines, such as those developed for COVID-19, exemplify the therapeutic potential of RNA technologies. These vaccines, including the mRNA-based Pfizer-BioNTech and Moderna vaccines, have demonstrated unprecedented efficacy and safety, setting new benchmarks for vaccine development^[12]^. Despite these advancements, the implementation of genetic therapies faces several challenges. Off-target effects remain a significant concern in CRISPR-Cas9 applications, as unintended edits could lead to deleterious consequences. Efforts to improve the specificity of CRISPR systems, such as developing high-fidelity Cas9 variants, are ongoing. Additionally, the efficient delivery of genetic therapies to target tissues poses another hurdle. Viral vectors, such as adeno-associated viruses (AAVs), are commonly used for delivering genetic payloads; however, their limited carrying capacity and potential immunogenicity necessitate the exploration of alternative delivery platforms. Lipid nanoparticles (LNPs) have emerged as a promising non-viral delivery system, offering advantages in terms of safety and scalability. LNPs have been successfully employed in RNA-based therapies, including COVID-19 vaccines, highlighting their potential for broader applications^[13,14]^.

### Psychological and lifestyle challenges

Inherited aortopathies, such as Marfan syndrome, Loeys-Dietz syndrome, and familial thoracic aortic aneurysms and dissections, impose a unique psychological toll on affected individuals. The initial diagnosis frequently triggers anxiety and fear, particularly concerning the risk of aortic dissection or rupture. The need for regular medical monitoring and imaging and the anticipation of potential surgical interventions often exacerbates this anxiety. In a survey of individuals with Marfan syndrome, 68% reported high levels of health-related anxiety, particularly surrounding imaging results and the possibility of sudden cardiac events^[22]^. Another study noted that up to 50% of patients with inherited aortopathies exhibited symptoms consistent with clinical depression, often linked to feelings of vulnerability and loss of control over their health^[12]^. The psychological distress is not limited to the affected individual but extends to their families, given the hereditary nature of these conditions. Concerns about genetic transmission to offspring often weigh heavily on family planning decisions. Studies have shown that individuals with inherited aortopathies frequently experience guilt and distress about the potential impact of the condition on their children, complicating decisions regarding reproduction and family dynamics^[13]^. Genetic counseling is critical for many, providing valuable information about inheritance patterns, reproductive options, and prenatal testing. A recent analysis highlighted the positive impact of genetic counseling in reducing anxiety and empowering individuals to make informed decisions about family planning^[14]^. Social challenges further complicate the experience of living with inherited aortopathies. The visible physical characteristics associated with syndromic conditions, such as Marfan syndrome’s tall stature and joint hypermobility, often draw unwanted attention. This can lead to feelings of embarrassment, social withdrawal, and stigma. A qualitative study involving adolescents with Marfan syndrome revealed that 70% had experienced bullying or negative social interactions related to their physical appearance, contributing to diminished self-esteem and social isolation^[15]^. The social stigma associated with physical features can also affect peer relationships and career opportunities, further impacting quality of life. Lifestyle modifications, including physical activity restrictions, dietary changes, and adherence to medical regimens, add another layer of complexity to the psychosocial experience of patients with inherited aortopathies. Physical activity restrictions are a significant source of frustration, especially among younger individuals. Many patients report feelings of exclusion and disappointment from being unable to participate in sports or other physically demanding activities. One study found that 62% of adolescents with Loeys-Dietz syndrome reported a negative impact on their social lives due to these restrictions^[16]^. Adherence to lifelong medication regimens and regular medical check-ups further disrupt routines, often leading to medication fatigue and financial strain. The interplay between psychological distress and lifestyle challenges often results in a diminished quality of life. Patient-reported outcomes consistently highlight the need for integrated care that addresses these conditions’ physical and emotional aspects. Cognitive-behavioral therapy (CBT) has emerged as an effective intervention for managing anxiety and depression among patients with inherited aortopathies. A randomized controlled trial demonstrated that individuals undergoing CBT reported significant improvements in anxiety and coping skills compared to those receiving standard care^[17]^. Similarly, mindfulness-based stress reduction (MBSR) programs have shown promise in enhancing psychological resilience and reducing stress among individuals with chronic illnesses, including inherited aortopathies^[18]^. Support groups and peer networks are crucial in addressing patients’ social and emotional needs. Connecting with others with similar experiences fosters a sense of belonging and validation. Peer mentoring programs, where experienced patients guide newly diagnosed individuals, have been particularly beneficial. Participants in these programs often report improved emotional well-being and greater confidence in managing their condition^[19]^. Online support groups and virtual communities have expanded access to peer support, enabling individuals to share experiences, exchange information, and provide mutual encouragement regardless of geographic location. Family-centered interventions are equally important, given the familial implications of inherited aortopathies. Family therapy and psycho-education sessions help improve communication, foster understanding, and promote collaborative decision-making within families. A study on family therapy for genetic conditions found that families who participated in therapy reported stronger bonds, reduced conflict, and improved coping mechanisms^[10]^. These interventions also address the unique challenges faced by siblings, who may experience feelings of neglect or resentment due to the attention focused on the affected family member. Advocacy organizations and community-based initiatives contribute significantly to improving the quality of life for individuals with inherited aortopathies. Organizations like The Marfan Foundation and the Loeys-Dietz Syndrome Foundation provide a platform for raising awareness, promoting research, and advocating for patient rights. These groups organize educational workshops, awareness campaigns, and community outreach programs to empower individuals with knowledge and resources to navigate their conditions better. A recent evaluation of advocacy efforts highlighted the role of these organizations in reducing stigma, enhancing social inclusion, and fostering resilience among patients and families^[11]^. Research on patient-reported outcomes emphasizes the importance of integrating psychosocial support into routine care for individuals with inherited aortopathies. Studies have shown that comprehensive care models, which include mental health services, genetic counseling, and lifestyle management, lead to better overall outcomes. For instance, a longitudinal study tracking individuals with inherited aortic conditions found that those receiving integrated care reported higher satisfaction, better emotional health, and improved adherence to medical recommendations^[12]^. Such models also recognize healthcare providers’ role in addressing patients’ emotional needs, highlighting the value of empathetic communication and shared decision-making.

## Concluding remarks

Genetic factors are integral to aortic health and the pathogenesis of inherited aortopathies, including Marfan syndrome, Loeys-Dietz syndrome, EDS, and FTAAD. Understanding the genetic basis of these conditions remains critical for accurate diagnosis, effective risk stratification, and personalized management strategies. Advances in genetic testing technologies and genomic research have greatly enhanced early detection capabilities, facilitated targeted therapeutic approaches, and provided essential insights for genetic counseling. These developments represent transformative steps toward improving patient outcomes and the overall quality of care for individuals and families affected by inherited aortopathies. Future research should prioritize the establishment of standardized genetic screening guidelines to identify at-risk individuals efficiently and reliably.

Additionally, efforts to discover novel genetic modifiers, biomarkers, and therapeutic targets are essential for refining risk prediction models, optimizing disease management protocols, and improving long-term prognoses. This research can also guide the development of innovative therapies that address the molecular mechanisms underlying aortic disease progression. For clinicians, integrating genetic testing into routine practice is a pivotal step toward achieving personalized care. Comprehensive diagnostic workflows incorporating genetic testing, imaging, and clinical evaluation will enhance early detection and facilitate timely interventions. Multidisciplinary collaboration involving geneticists, cardiologists, surgeons, and psychosocial experts is essential for implementing holistic, patient-centered care strategies. Clinicians must also advocate for increased access to genetic testing and counseling services to ensure equitable care, particularly in underserved populations. Enhanced awareness, education, and advocacy efforts are critical to reducing stigma, promoting early intervention, and fostering a supportive environment for individuals with inherited aortopathies. Policymakers and healthcare organizations should collaborate to develop public health initiatives, educational campaigns, and funding mechanisms supporting research, healthcare delivery, and patient advocacy. By addressing these challenges collaboratively, the medical community can advance the field of aortic health, improve clinical outcomes, and empower individuals with inherited aortopathies to lead healthier, more fulfilling lives.

## Data Availability

This published article and its supplementary information files include all data generated or analyzed during this study.
